# Pain Outcomes with an Elliptical Regimen (POWER) Study: Identifying the Proper Dosage of Exercise for Therapeutic Effect in Persons with Chronic Back Pain

**Published:** 2020-04-29

**Authors:** Timothy Dillingham, Jessica Kenia, Adrian Popescu, Christopher Plastaras, Scott Becker, Frances Shofer

**Affiliations:** 1 Department of Physical Medicine and Rehabilitation, University of Pennsylvania School of Medicine; Philadelphia, PA, USA; 2 Penn Therapy and Fitness Rittenhouse, Good Shepherd Penn Partners; Philadelphia, PA, USA; 3 Department of Emergency Medicine, University of Pennsylvania School of Medicine; Philadelphia, PA, USA

**Keywords:** Back Pain, Exercise, Elliptical trainer, Kilocalorie, Physical therapy, Aerobic

## Abstract

**Background context:**

Exercise therapy for low back pain has long been prescribed as one of the initial remedies for back pain. Traditional therapy is completed under a therapist’s supervision and consists of lumbar stabilization, aerobic exercise and stretching exercises. Recent studies have explored treating back pain with aerobic exercise such as walking which can be done anywhere and without supervision which is lower cost and easily administered.

**Purpose:**

To assess a therapeutic dosage of aerobic exercise that is associated with pain reduction in persons experiencing low back pain.

**Study design:**

Case series.

**Participant description:**

Sixteen patients entered the study and twelve patients completed the study (mean ± SD: age 51 ± 11 years; weight 89.2 ± 16 kg). Subjects were included if they were ages 18–65, had chronic back pain lasting for more than 3 months and a score of greater than 30% on the Oswestry Low Back Disability Questionnaire.

**Methods:**

Subjects underwent a six-week exercise program using the elliptical trainer three times each week. Exercise duration was steadily increased each week for the length of the study. The total cumulative amount of work that coincided with significant reductions in chronic low back pain was then identified.

**Results:**

At 4 weeks, pain scores were significantly reduced from baseline (3.2 vs 4.7, p<0.0001). This significant pain reduction corresponded to an average of 30.8 Kcal/kg of body mass in cumulative work performed. Pain was significantly reduced by 21% and 32% on the Oswestry Questionnaire and the PROMIS 29 respectively.

**Conclusions:**

These pilot findings suggest that approximately 30.8 kcal/Kg of accumulated physiological work is a therapeutic “dosage” of exercise needed for significant reduction in chronic back pain. Clinicians can begin to use this benchmark for their oversight of rehabilitation programs to determine if an exercise program has been sufficiently intense and long enough in duration for managing their patients with chronic low back pain.

## Introduction

Low back pain (LBP) is one of the most prevalent conditions that will affect 70–85% of individuals at some point in their life [1]. LBP accounts for 2.3% of all visits to the physician, and is the most common area of the body to experience pain [2]. Exercise has been a mainstay for managing persons with chronic low back pain for almost 40 years [3,4]. These exercise regiments that have various components have been shown to be effective. Increasingly, recent evidence shows that less sophisticated aerobic exercise alone is effective at reducing LBP symptoms in persons with chronic LBP [5].

Implementation of exercise treatment plans for individual patients is challenging because there is no clear dosage of exercise with which to judge the proper intensity and duration of exercise and the point at which therapeutic effects can be expected. In short, we lack the most fundamental measurement for quantifying whether an adequate exercise program was provided — a therapeutic dosage of work done over the course of a prescribed rehabilitation program that is generally associated with LBP symptom resolution.

The purpose of this pilot study was to address this major gap in our knowledge about therapeutic exercise. Specifically, we sought to determine if a clear therapeutic quantity of exercise (work) exists that is associated with significant LBP reduction. This information would then inform a larger scale prospective trial using aerobic exercise. This avenue of investigation is particularly timely given the widespread opiate crisis and the CDC Opiate guidelines which underscore the importance of rehabilitation as a first line treatment for persons with chronic pain instead of opiate medications [6].

## Materials & Methods

### Participants and setting

The study was approved by the University of Pennsylvania IRB committee. Subjects were recruited from an academic physical medicine & rehabilitation based spine clinic. Individuals that expressed interest in the study were screened for eligibility by a research assistant. Inclusion criteria were; i) age 18–65, having chronic back pain lasting three months or greater, and ii) a score of 30 points or higher on the Oswestry Low Back Disability Questionnaire [7]. A score of 30 or more reflects a level of pain and disability that was more severe and clinically severe enough to seek treatment. Exclusion criteria included: i) previous spine surgery within the past 6 months, ii) radiological findings such as fractures or infection, iii) disc herniation with an active radiculopathy or spinal stenosis, iv) widespread myofascial pain, v) inflammatory joint disease, vi) chronic opiate medication use, vii) spondylolisthesis grade II or more, viii) myelopathy, ix) neoplasia, x) cauda equina syndrome, xi) pregnancy, xii) severe orthopedic issues, or xii) obvious ongoing psychiatric illness. Each eligible participant filled out an American College of Sports Medicine (ACSM) Health Screening Questionnaire [8] to determine whether physician clearance was needed prior to entering an exercise program. Clearance from their primary physician was obtained when needed. Participants were all consented by a Research Coordinator.

### Measurement

The Oswestry Low Back Disability Questionnaire was administered prior to participation, during several two week intervals of the study, and at the end of the trial. The Patient-Reported Outcome Measurement Information System (PROMIS 29) [9] questionnaire was also completed pre and post treatment to assess anxiety, physical function, depression, satisfaction with social roles, fatigue, sleep disturbance, pain interference and pain intensity. Before and after each exercise session, the participant was asked to mark their pain level on a 10-point Numeric Pain Rating Scale (NPRS) [10] which is a segmented numeric version of the Visual Analog Scale (VAS) where the participant selects a whole number (0–10) that best reflects the intensity of his/her pain. The average of these two scores over three visits were used for the weekly pain analyses. The time duration, kilocalorie (kcal) and distance were recorded after each elliptical exercise session. The total amount of kcals were calculated by adding together the kcal from each session. This cumulative amount of work was then analyzed statistically along with the weekly pain scores.

### Equipment

We chose an elliptical trainer for exercise in this study because it is a relatively low impact exercise machine that requires energy expenditure similar to a treadmill and also utilizes upper body muscles [11]. An elliptical trainer incorporates the torso and core musculature and can provide quantitative kcal information for each exercise session. Our elliptical trainer machine featured moveable handles for upper body strengthening. The resistance was increased weekly to accommodate and promote the conditioning effect expected for patients over the course of the six-week trial.

### Procedures

Participants completed supervised exercise sessions using the elliptical trainer three times a week for a period of six weeks. If a session was missed, it could be made up at the end so that 18 total sessions were completed. Participants were encouraged to complete all of their sessions as scheduled to achieve the best results. They were instructed to refrain from any other types of physical therapy for their back pain during the study duration. To obtain a baseline of the participant’s fitness they were asked to complete a two-minute warm up, then continue for as long as possible up to 15 minutes. They were told to achieve a “moderate” pace using the RPE (rating of perceived exertion) scale so that they achieved a 6 on a scale 1 (very light exertion) to 10 (maximal effort). They were instructed to stop at the 15-minute mark as we did not want to exacerbate any symptoms. For the majority of participants, this was their first exposure to the elliptical machine and they could only complete 5–10 minutes of exercise on the first visit. An important aspect of this trial was that the exercise intensity was steadily increased over the six weeks. Five minutes were added to the individuals’ exercise duration each week. A two-minute warm up and a two-minute cool down period were included to gradually raise and lower the heart rate. Each participant’s age, weight and gender were entered into the elliptical machine to obtain an estimated caloric (kcal) expenditure for each exercise session. The same elliptical machine was used for each participant in the study. The elliptical kilocalorie algorithm was used to capture kcal expenditure data, and although it may not be the most accurate method, the kilocalories recorded were consistent across participants. The distance, time and caloric expenditures were documented after each session. Our outcomes of interest were pain intensity and cumulative exercise work (kcal) performed by the subjects over the course of the six weeks.

### Analysis

The Oswestry Back Disability Questionnaire was administered prior to the study and at two week intervals during the study, while the PROMIS 29 was completed pre and post treatment. Pain was measured on a 0 through 10-point numeric pain rating scale before and after each exercise session. These scores for a week were averaged to yield the weekly score that was used in statistical analyses. To assess changes over time in pain, Oswestry scores, and kcal burned, analysis of variance in repeated measures was used. Tukey-Kramer t-tests were used to adjust for multiple pairwise comparisons. To assess changes in the PROMIS 29, the paired t-test was used. All analyses were performed using SAS statistical software (version 9.4, SAS Institute, Cary NC). A p-value <0.05 was considered statistically significant.

### Sample size calculation

As this was an exploratory study, a 1–2 unit change in pain level was considered a clinically meaningful difference. We estimated an 80% power with alpha set at 0.05 for a 2-tailed test, approximately twelve participants were predicted to detect this difference.

## Results

Sixteen participants were consented and enrolled in the study. Of this initial sample, twelve participants (five males and seven females) were able to complete the study. Their ages were a mean of 50 ± 11.2 years. Four participants withdrew over the course of the study. Two were due to unrelated medical issues; one participant developed a respiratory illness that prevented her from exercising for two weeks, while the other broke his shoulder and could not exercise safely on the machine. The last two gave reasons of time restraints and inability to attend the sessions three times per week as required by the protocol. No adverse musculoskeletal or cardiovascular events occurred and patients were able to tolerate this program, although two patients did mention slight discomfort in the knee and ankle joints which was not enough to prevent continuation in the exercise trial.

**Figure 1:**
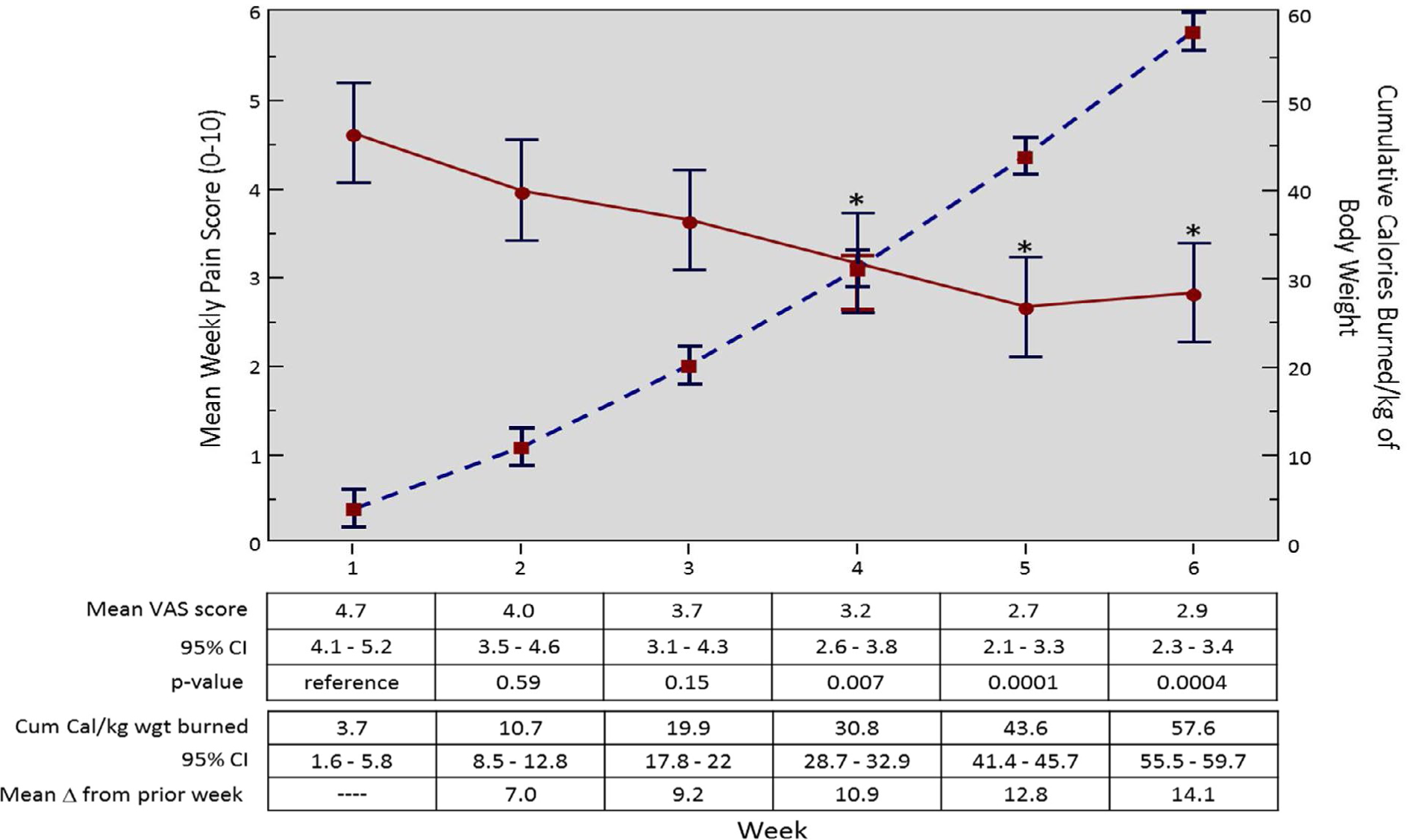
Mean weekly pain over six weeks and cumulative kcal burned per kg of body weight.

By week 4, there was a significant decrease in average weekly pain score compared to week 1 (3.2 vs 4.7; difference =1.5, 95% CI: 0.6 −2.3, p=0.007); and this improvement remained significantly different from week 1 for the duration of the study (week 5 and 6, pain score=2.7, 2.9, p=0.0001, 0.0004, respectively (Figure 1)). Pain reduction corresponded with 2,617 cumulative kcal, or an average of 30.8 kcal per kg of body weight. Average kcal burned significantly increased throughout the study period, with consistently larger increases each week.

Similarly, the Oswestry Low Back Disability Questionnaire showed reductions from 43% disability to 32% at week 4, and 33% at the conclusion of week 6 (both significant at p < 0.005). The only significantly difference on PROMIS-29 was the pain intensity score subscale, which dropped from 5.6 pre-treatment to 3.8 post-treatment (p<0.005).

## Discussion

In this prospective pilot trial, aerobic exercise performed on an elliptical trainer significantly reduced pain as indicated by the Oswestry Low Back Disability Questionnaire, Numeric Pain Rating Scale and PROMIS 29 pain intensity score, when a total dose of cumulative physiological work reached a level of 30.8 kcal/kg of body weight. This was the cumulative exercise achieved over four weeks of exercise with a regimen that continuously increased the intensity of the workout sessions with a focus on exercise and not symptoms. These results suggest why shorter exercise programs of two or three weeks are less effective — insufficient work is done in these shorter programs. These results are intriguing and suggest that a clinically measureable cumulative amount of total body exercise (30.8 kcal/kg) provides a dosage of aerobic exercise associated with significant pain reduction for persons with chronic LBP.

Although a pilot trial, the reduction in pain across three outcome measures was compelling. This suggests that clinicians can begin utilizing a cumulative work kcal measure to evaluate a patient’s exercise program. If the total work is less than 30.8 kcal/kg for patients undergoing physical rehabilitation for LBP they should keep exercising. Such quantifiable information could well find a use with mobile exercise monitoring devices such as the FitBit, or the iPhone. A supervised exercise program has the greatest chance of compliance and now with a target work level, patients can more actively work towards a quantifiable exercise therapy program goal.

One limitation of the study is that our caloric expenditure information was derived from the elliptical exercise machine. In a larger scale study, we intend to use data from exercise machines as well as heart rate monitors to provide more means of estimating total work performed. Another limitation is that only one method of exercise was available to the subjects, the elliptical trainer. We chose this to minimize joint stresses. The elliptical trainer also used the arms to exercise and for this reason these upper body muscle groups contributed to the overall calorie levels of work performed. These findings, although compelling, do not tell us whether exercise at a low intensity for a longer period of time than 6 weeks will result in the same pain reductions as a higher intensity exercise program for shorter (4 to 6 week) exercise regimens.

This pilot study had notable strengths that suggest these findings are useful and generalizable. The significant differences found across three outcome measures with a sample size of 12 suggests that the observed pain reductions were clinically meaningful and significant treatment effects. The degree of pain reduction across these three outcome measures reflects clinically meaningful changes. None of the patients dropped out because of a new overuse injury from the elliptical exercise regimen itself. The total work done during each exercise session was obtained from the elliptical trainer and added to the previous session’s total work accomplished during the exercise program. These kcal totals correlated so well with pain reduction, that this may be the easiest and most clinically useful means of tracking patient progress in their therapy program.

Insurers will frequently limit duration of exercise for beneficiaries. This study provides evidence that a minimum amount of total work needs to be accomplished and that therapy programs must extend for 6 weeks or more for patients to reach the threshold of exercise (30.8 kcal/kg) needed for optimal therapeutic effect.

Once back pain has become chronic, a variety of therapies may be prescribed for pain relief. Active therapy has been found to be more effective for pain reduction in both short term and long term versus passive modalities such as ultrasound, massage and heat [5,12–19]. Physical therapy treatments vary widely from more passive modalities, to lumbar stabilization, stretching, and aerobic exercises. Treatment of chronic low back pain varies across different therapists. One study revealed that almost all therapists reported using at least one treatment classified as having limited evidence of efficacy [20].

Aerobic exercise interventions can reduce low back pain as effectively as strength training [21] and physical therapy [22–24], and have the significant advantage over passive modalities such as TENS, ultrasound and heat [5]. What is clear from previous studies is that exercise is an effective means of addressing back pain. What is not known from previous investigations is the proper dose (reflecting intensity and duration) of exercise required to ensure a high probability of symptom reduction.

Patients on exercise programs may not achieve the intensity or duration to affect changes in their pain. Clinicians often make judgements about exercise and conservative care “failures” when in fact these ‘failures” might simply be due to insufficient total work was performed — a sub-therapeutic dosage of aerobic exercise. Failure of conservative therapy is often utilized when advocating for surgical interventions for spine pain. Having a *quantitative* means of assessing a patient’s participation in a physical therapist’s supervised exercise program would be of great practical use on many levels.

Such quantitative information would be useful in advocating for insurer reimbursement for a course of therapy lasting 6 weeks or longer which may then reduce long term healthcare expenditures and subsequent surgical interventions. Spine surgery is often undertaken after a determination that a conservative program “failed”. The rate of back surgery in the United States was at least 40% higher than in any other country and was more than five times those in England and Scotland [25]. These high rates of surgical interventions in our healthcare systems underscore the need to be precise in our conservative exercise care for persons with chronic LBP. Clinicians and therapists must ensure that the optimal exercise program was in fact, achieved before determining that such a program “failed”.

The elliptical trainer is suitable for most levels of fitness, and may be an effective means to reduce LBP based upon our findings. These findings are in agreement with other studies that showed clear improvements in LBP with active exercise [21–24]. Murtezani and colleagues compared two randomized groups of 101 low back pain patients [5]. Half were placed in an aerobic exercise group which consisted of using the treadmill, bike or stairclimbing for three days per week; while the other half received passive therapies such as TENS, heat and ultrasound. After 12 weeks, significant reductions were found in all outcome measures for the exercise group, versus no significant differences in the passive modality group. These results by Murtezani et al, along with ours suggest that it might not be the type of aerobic exercise used, but rather the cumulative exercise level reached over the duration of the program.

In our study, at the four-week mark, the average exercise session length was about 26 minutes. A target of 30 total minutes of exercise on the elliptical training machine each session is reasonable based upon our study results where all but one participant achieved this duration of exercise by the end of the study (six weeks). A larger scale study is needed to fully explore and confirm these findings regarding the proper dosage of exercise to achieve pain reduction. Nonetheless, these findings can be useful in clinical settings now and give therapists and clinicians valuable information regarding whether or not a conservative rehabilitation program was sufficient in duration and intensity of exercise to effect pain reduction. Although these findings are preliminary, this exercise threshold — 30.8 kcal/kg of cumulative exercise — gives therapists and patients a goal to strive for in a conservative exercise-based rehabilitation program.

## Abbreviations:

LBP: Low Back Pain
CDC: Center for Disease Control
IRB: Institutional Review Board
ACSM: American College of Sports Medicine
PROMIS: Patient Reported Outcome Measurement Information System
NPRS: Numeric Pain Rating Scale
VAS: Visual Analog Scale
RPE: Rating of Perceived Exertion
TENS: Transcutaneous Electrical Neural Stimulation
